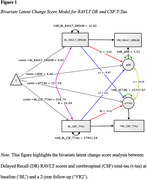# RAVLT Delayed Recall predicts two‐year changes in cerebrospinal fluid total‐tau for cognitively healthy older adults

**DOI:** 10.1002/alz70861_108818

**Published:** 2025-12-23

**Authors:** Lauren E Sather, John L. Woodard, J. Carson Smith, Kristy A. Nielson, Sally Durgerian, Audrey Zhu, Maria Khrestian, Stephen M. Rao

**Affiliations:** ^1^ Wayne State University, Detroit, MI USA; ^2^ University of Maryland, College Park, MD USA; ^3^ Medical College of Wisconsin, Milwaukee, WI USA; ^4^ Cleveland Clinic, Cleveland, OH USA

## Abstract

**Background:**

Alzheimer’s Disease (AD) is a disease that continues to increase in prevalence. Given its burden and cost, preclinical detection of AD is crucial. Neuropsychological tests are easily administered, non‐invasive techniques that can aid in detecting Preclinical AD. This study investigated whether baseline delayed recall (DR) scores on the Rey Auditory Verbal Learning Test (RAVLT) may predict two‐year changes in cerebrospinal fluid (CSF) total‐tau (t‐tau), a hallmark AD biomarker.

**Method:**

149 cognitively healthy older adults (M*_age_* =70.3, 75 APOEε4 carriers and 74 APOE ε4 non‐carriers) were included in this study. As part of a more extensive longitudinal study, participants underwent testing at baseline and a two‐year follow‐up period. Participants were administered the RAVLT, and CSF t‐tau was collected at both timepoints via lumbar puncture. Bivariate latent change score models were utilized to investigate the possible predictive relationships between RAVLT DR scores and CSF t‐tau.

**Results:**

Carriers and non‐carriers did not differ significantly on any parameter; therefore, the pooled data were used for analysis. Participants’ RAVLT DR scores (*M*=4.5, *z*‐score=5.5, *p* <.001) and CSF t‐tau (*M*=157.3, *z*‐score=3.3, *p* <.001) increased significantly from baseline to the two‐year follow‐up. Baseline RAVLT DR scores were negatively associated with cognitive change over two years (*β1*=‐.43, *z*‐score=‐6, *p* <.001). Similarly, baseline CSF t‐tau level was negatively associated with tau change over two years (*β2*=‐.20, z‐score=‐2.8, *p* =.003). Baseline RAVLT DR scores predicted two‐year t‐tau change (*γ1*=‐8.9, *z*‐score=‐2.2, *p* =.014) indicating that participants who had higher baseline scores on RAVLT DR exhibited less change in CSF t‐tau over two years. Baseline CSF t‐tau level did not predict two‐year RAVLT DR changes (*γ2*=‐0.002, *z*‐score=‐1.4, *p* =.09).

**Conclusion:**

Our findings showcase that baseline RAVLT DR performance is a leading indicator of CSF t‐tau change over two years in cognitively health older adults. Notably, baseline CSF t‐tau level did not predict cognitive change over time. Given the RAVLT’s low‐cost and ease of administration, it has considerable promise for early prediction of subsequent AD‐related fluid biomarker changes later in the disease course.